# Concerning the Possibilities in the Art of Filling Teeth

**Published:** 1899-06

**Authors:** H. A. Smith

**Affiliations:** Cincinnati, Ohio.


AMERICAN JOURNAL

—OF—

DENTAL SCIENCE.

VOL. XXXIII. BALTIMORE, JUNE, 1899. No. 2.

### Selections.

ARTICLE I.

### CONCERNING THE POSSIBILITIES IN THE ART OF FILLING TEETH.

H. A. SMITH, D. D. S., CINCINNATI, OHIO.

I have been led to this subject—Possibilities in the Art of Filling Teeth—by observing from time to time, on the part of not a few of our more thoughtful and experienced practitioners a feeling of distrust or disappointment in their ability to save teeth by the operation of filling.

This feeling of distrust was given expression by one of our most skillful practitioners at the last meeting of the Ohio State Dental Society, when he said, "I think filling teeth is a failure." Another practitioner of experience and reputation, in Chicago, recently stated publicly, "that quite one-third of his time was occupied in refilling teeth, mostly approximal surfaces of bicuspids and molars, that had not been filled for more than two years." These and like expressions which we often hear, coupled perhaps, with feelings of chagrin at our own frequent failure to permanently benefit our patients, may well cause us to pause and inquire concerning the possibilities in this department of our practice.

The art of filling teeth has been followed for more than one hundred and fifty years. Although filling is the true therapeutical treatment for dental caries, during most of this long period, the practice was somewhat empirical. With the erroneous views held as to the active causes of caries, the operation of filling teeth was based upon observation and experience alone. As practiced now, filling has a true scientific basis.

It must not be forgotten that there are two objects in filling teeth; the arrest of caries and the prevention of its recurrence. Both of these objects must be kept clearly in mind when we consider the possibilities in filling teeth.

The arrest of caries is, as a rule, not difficult. We can accomplish this by removing the active causes of caries, surgically and chemically. But since caries would not long remain inactive following excavation and sterilization we are compelled to resort to filling. With the cavity prepared according to approved rules, and with the use of gold skillfully manipulated, we may, in favorable cases, hermetically seal the cavity. And again we say, caries is arrested.

If the cavity is upon the occlusal surface and we have sealed it so that water, fermentable food debris and micro-organisms cannot enter, we need not concern ourselves about the recurrence of caries. If, however, the cavity is upon the approximal surface, where the teeth are most given to caries, and the operator has only in mind the arrest of caries, the life of his filling, no matter how skillfully made, will not, as a rule, exceed the two years' limit mentioned by our Chicago friend. In cases where predisposition to caries is strong, a close study and judicious application of the *gospel of extension for prevention* is essential for the preservation of the teeth. Prof. Black has written ably upon the whole subject, both scientifically and practically. Upon this point he says, "Whenever the predisposition to caries is found to be strong, this fact calls for the utmost diligence in planning the defence of the teeth, and especially in laying out the lines of the enamel margins of cavities in such positions that they will be well cleansed by the excursions of food over them in mastication. For this purpose the buccal and lingual enamel margins of approximate surfaces should be carried well out from the contact point, and the form of the occlusal surfaces so shaped as to direct the excursions of food well into the buccal embrasures of the interproximate space to facilitate the continuous cleansing of these margins." "With the best technique of our time, the margins of filllings must be regarded as danger lines."

In describing the so-called ideal filling, I have not described an impossible operation. Such fillings are being made by hundreds of dentists to-day. These operations are only possible, however, to the skillful, painstaking, conscientious and heroic dentist. Such men are scientific as well as eminently practical. They are naturally, and by right ought to be, representative of their profession, and that which they accomplish in filling should be regarded as the best expression of the art. Yet operations from the hands of our best men fail over and over. But since the last resources of the art have been exhausted in making these so-called failures, they represent in a negative way, the possibilities in filling teeth. As a rules our failures attract more attention than our success in filling. This is unjust. We should be judged by our successes.

I do not wish to be understood as taking a pessimistic view of the operation under consideration. Filling teeth has been of vast benefit to our race, and will continue to be. Always, when teeth are filled by a skillful and trained operator who exercises his best judgment in the case, the patient receives benefits worth all the services cost. Unfortunately, the people have been led all along to believe that filling was an infallible remedy for carious teeth. This is no fault of the people, but is due to the ignorance and cupidity of a certain class of practitioners.

Mr. Charles Tomes says: "There is, perhaps, no other operation performed upon the human body which is attended with the same unqualified success as that of filling teeth, for we not only succeed in the majority of cases in arresting the further progress of disease, but we also replace the part which has been lost by an imperishable material, and render the organ as useful as it was prior to becoming the subject of caries. It is however, a great error to suppose that filling will, under all circumstances, permanently save the tooth, even in cases which at the time the operation promised favorably." "There are those who are disposed to regard the decay of a tooth which has been filled as the result of the want of skill or of care in the operator. Such an opinion is perfectly untenable when the character of the operation is considered in connection with the issues which are involved, and the various conditions under which disorganization may be effected." "There are other sources of failure than the assumed want of skill in operating, and such are not under the control of the dental surgeon or of the patient."

Notwithstanding the more recent investigations of Miller, Black, Williams, Andrews and of Mr. Tomes himself, all of which investigations have a practical bearing upon filling teeth, the above quotation accurately describes the situation to-day so far as the possibilities of the art are concerned.

Objection may also be made that I have only referred to one kind of filling material—gold—when in a good number of cases the possibilities of filling are best accomplished with other materials. While this is true, it does not do away with the fact that, when its use is indicated, gold is the very best material known to us.

Of amalgams, I may say, in my practice they have been disappointing. The newer amalgams formulated by

Professor Black, called "quick-setting," I have used to a limited extent, and in my opinion they promise to be an improvement over most of the amalgams now in use.

It may be interesting to inquire in this connection what would be the effect upon the practice of filling teeth if the ideal filling material should be discovered? I venture the opinion that no more perfect fillings would be made than now. Naturally, because of easier manipulation, the number as well as the proportion of good saving fillings, would greatly increase. Given the same unfavorable environment, the perfect filling made with the ideal material would not last any longer or preserve the tooth any better than the perfect filling made to-day of gold.

In future if the operation of filling teeth shall meet the reasonable expectations of patients, more study and thought must be given by the dentist to the prevention of caries. Prophylaxis should at last receive attention equally with the mechanical technique of the art.

DISCUSSION.

Dr. D. A. House: Some have made the statement that our best operators have reached the limit in skill and perfection in the filling of teeth, and further improvement must come in the changes of environment, which will lesson the recurrence of decay. It is not my intention to discuss the methods—to improve the environment. To increase the amount of oral secretion would prevent it.

I will consider whether the possibilities in the art have been reached. Concerning the question "Is the filling of teeth a failure?" it is no more applicable to us than that the science of medicine is a failure. A body is always liable to the recurrence of disease. Because a great number of teeth fail in the lapse of two or three years is not a rule that filling teeth is a failure. It is usually due to the carelessness of the operator. The work of some of our best operators is still open to criticism. Not so in every filling. Many of the smallest, most difficult to make, reach a point which cannot be excelled by our present filling material. While I acknowledge and practice the desirability of keeping the enamel margins out of point of contact, yet many operators cease before this has been reached because of pain to the patient. So long as the dentist is required to stand this strain he will certainly become tired and nervous and so much overtasked that it will be impossible to do his best work under these conditions. If it were possible to charge sufficient for the work so as to make as good a living within five hours of a day as he does now in ten, the quality of work would no doubt be much improved. Does it not often occur to the operator "Oh, I'm not getting much for this," so I will make it accordingly. This makes poor fillings.

Another thing is the fear of becoming known as slow operators. Those known in the profession as best and most skillful are the most rapid. These things often make the work a failure. I have known good operators to spend half a day on one filling. There is no doubt that rapidity is often a source of failure in operations. Therefore until we get better prices for our work and put more time on fillings and are able to cut living dentine without torturing patients, the possibilities in the art of filling have not been reached. [Loud applause].

Dr. Barrett: I am not going to offer any commendations on this paper. "That life is long which answers life's best ends." That paper is long which most completely covers the subject in the fewest words. Although quite a complete paper, there are yet a few facts which have not been fully utilized.

I want to refer to a few of the difficulties which must be overcome in the insertion of the filling.

It has been positively determined that caries is due to the solution of the calcic salts through which the action pronounced by the proliferation of micro-organisms, these coming in through the opening in the enamel, or because of some imperfection in the enamel. This is followed by a complete breaking down with the collection of many organisms. We call this the field of infection. Suppose we have a cavity which is just below the point of contact with the next tooth, at which decay usually has its inception. The reason, it is just the point at which food lodges, which becomes the matrix in which the organs proliferate with the production of an acid, and hence we have below this point the beginning of decay and solution of enamel.

The usual process of preparing a cavity before filling is to excavate as much of cavity as is disintegrated; but you saw in the slides that beyond this portion we had the zone of infection or commencement of decay, and which precedes the breaking down in the cementum, in the openings called the inter-tubular spaces. These enlarge and two spaces will unite into one, break down and the zone of infection will enlarge, and this continues unless checked. I do not suppose that a cavity is completely excavated when we reach inward near the limit of the zone of infection. Suppose I excavate and prepare my cavity, as most dentists do, have I reached the point of danger ? Will an ordinary filling of this kind prevent the tooth from decay, and the entrance of the organisms which we know and which we all are the cause of dental caries ? If we know the absolute cause, can we remove the last predisposition to decay ? Yes, if it were possible to reach to the limit of this zone of infection, and if at the same time we can so reduce the environment as to prevent further decay. Dr. Black has thoroughly demonstrated this, and it is so strange that so many think with their elbows and do not comprehend the fact.

I want to make a point right here, and that is that this zone of infection has gone beyond the point at which excavation of the tooth would in almost any case endanger the pulp and destroy the vitality of the tooth. It is impossible to excavate back to the point of infection, and yet as long as there is that zone of infection in which has been implanted all the micro-organisms which will produce the caries, and everything necessary to carry it on, there is danger of the filling becoming a failure. We have conditions which will cause re-decaying. That is the great trouble and danger; we want to overcome it if possible.

We are sometimes asked "What does all this mean about the bug theory of caries? There is a practical use of this. All of these organisms which produce this disease are called ærobic—that is, live only in the presence of oxygen. If imbedded in tissue, where oxygen could not penetrate, they could no longer exist. We can artificially produce this, and hence when we have thoroughly, hermetically sealed that cavity from oxygen or the air, we have destroyed these organisms, and that is why a filling must be absolutely perfect if it is going to preserve the tooth; not the slightest chance for the entrance of bacteria. Air must be excluded and the cavity should be hermetically sealed.

Gold is not the best material with which to hermetically seal a cavity, but it possesses other qualities which make it the best filling material. Gutta-percha is probably the best material for preventing recurrence of decay, but it requires a very high degree of skill to put in a filling of this material. We can use some antiseptic which will penetrate the dental tubuli and thoroughly destroy the gums, hence the necessity of thoroughly sterilizing a cavity before commencing a filling. I do not pose as the highest authority on filling teeth. There is something else which I think necessary—the use of something more penetrating—which will protect the tooth from recurrence. I use carbolic acid, and then I cover over that portion which I think contains the infected dentine some kind of material before inserting the filling. I use a solution of some of the balsams—Canada balsam or something else. I spread this over the infected area and it assists in preventing the penetration of air to this point to cause proliferation of micro organisms.

After determining how far has been the penetratium of micro-organisms and excavating as much as possible, the next thing is to thoroughly sterilize it by soaking well with some thorough antiseptic, then drying and covering the affected area with something to exclude air, and over this insert filling. Even then you have not a perfect filling. After having gone over all this the next thing is to see that the mouth is thoroughly antiseptic.

I have never found such a case as this, and I do believe that no such condition as this exists, as it is impossible to keep the mouth in complete antiseptic condition. The ordinary mother, for example, has something else to think about than care of the teeth. She does not give them her care, and consequently the time finally comes when she comes to you with her teeth in the worst possible condition. If she would use some mouth wash and thoroughly sterilize her mouth, recurrence of decay would not take place.

The chief factor is to be thorough in the sterilizing of the cavity and keep it so. If we do this there need be no fear of recurrence of decay.

Dr. Butler: Dr. Barrett has such a wonderful power of suggestiveness that it may not be within my power to have any effect upon him. But as Dr. Smith has said that many declarations that filling teeth is a failure have been made, I will say I am one who made such a statement, and I do not expect to use any argument to establish what I said as true. I said it with this view of the subject that, with all the improved methods of securing a tooth from moisture during the process of the operation and with what is supposed to be the most modern achievements in the way of instruments and preparation of gold, I question whether there are any more successes or as much following the methods in that direction to-day as there was forty or fifty years ago. Still, if that be true some will say that it is not so, but nevertheless look the case fairly in the face. Take the number of operators to-day and compare them with the number forty years ago, and the question is whether we make fillings that are any more saving to the tooth. If we are not doing so the question still comes up, is it a failure? We do not any of us question the operator's ability to fill a tooth, but it is the recurrence of decay. Dr. Smith has spoken of gold being the best material, and he has also mentioned some other materials. Gentlemen it is not the filling that fails; it is the tooth structure. That is the point and there is where our great effort should be put to save a tooth from destruction. That is the prime object of patients coming to us, and that should be our principal effort.

Dr. Barrett, I think, stated a complete and perfect gold filling would hermetically seal a cavity. With all our fine instruments and skill and book gold, etc., how many of us succeed? Take all the fillings made by the most skillful operators, put them under the magnifying glass and see what kind of margins a great many possess.

We have two structures to deal with—gold and enamel. I fear very few come completely up to the enamel, it is so easy to bruise and chip off pieces of enamel. Gold is soft and maleable, while enamel is hard and brittle, and that part which is elastic we bruise. How are we going to have thorough sealing of cavity? It is the most infinitesimal openings through which these micro-organisms pass.

Why is it that some of our old soft-foil manipulators with hand pressue put in fillings which stayed and kept tooth substance? The great power of tin lies in the fact that you can put it so close to the walls of the cavity that it seals the cavity and does not bruise the walls of same as under the constant hammer and hammer, that is necessary with gold.

Take our rapid operators, for example, who put in a very large filling in a very short time. I think it should take a half day to put in such a filling so it will save the tooth. Take this class of fillings, put in in a very short space of time and examine them in a year or two or more. Take the fillings out and you will see there is a certain layer of dentine under the filling which is devitalized. This comes from the over-packing of gold—excessive hammering. In such cases it is simply a matter of toleration how long that filling will be retained there. We have this tissue between gold and living tissue, which is dead, and it does not take long for moisture to creep in there and cause further decay.

"Gutta percha," so says Dr. Barrett, but it takes a good amount of skill to put in. It is also quite porous. Take it out of a cavity after having been in for a short time and hold in any flame and you will see it is porous—water will be driven from it. If placed in some essential oil before applying to the cavity it will exclude moisture to some extent, and also aid in sticking and cause it to stay in the cavity. It rolls out very readily also. It should be used only as temporary filling.

This talk about perfect fillings; they are perfect enough, but do they seal the cavity perfectly? That is the main point for us to consider. Everybody in these times tries to get hold of some of these golds which are so plastic and easily manipulated. Some of the older members of the profession when this gold first came in thought it was a marvel, and we would have perfect fillings. After a while we found these fillings were leaky, and patients came back to us. I just took the privilege of examining around the borders of some of the fillings made here, and I found they were very poor. If we pack the gold by hand pressure we must use great force. It is no trick to pack gold in mass, but it takes a good goldsmith to operate along the line of surgery, and we have some of the finest operators along that line. We should have some of the finest operators along that line. We should stick to fine-pointed instruments around all the borders.

Dr. Smith: Dr. Butler has stated he believes we fail. We are not discussing failures, but perfect fillings. He has said filling teeth was a failure before, and he said it was due to the fact that we did not get hold of the case early enough. What do you mean by that?

Dr. Butler: If I did make that statement I state it more emphatically. The smaller the cavity the less deeply we have to go into the tissue and the better the vitality of tooth. I doubt whether a tooth which is two thirds gold is going to remain there in as good shape as if it had one-half the amount of gold—if the pulp is alive. In the first place you have a larger area of margins to secure, and in the second place you have got a larger bulk of gold. I doubt very much if the tooth is as strong in the former case.

Dr. Cook, of Chicago: The point which struck me is this—the first cause of decay. Can we make a better tooth by filling than it originally was? If we have a fracture of radius it is not possible to have as good bone there as we had before fracture. Therapeutic measures, which is simply a filling, can be made perfect, and I believe very much in the statement of Dr. Butler, who said that the *tooth itself* did not last over two years. Dr. Black has recently been carrying on some experiments, which I have seen a great deal of, with filling material, and that is in regard to packing amalgam. The ideal filling lies in the plastic operation. We are not doing the gold work that was done by our older operators. The operation of packing the gold with mallet against the dentine is, to my mind, a very important one.

The bruises to the dentine are also important of consideration. I had the pleasure some years ago of seeing a filling made by Dr. Holbrook which had been in there for forty years. The patient was a women. She had had her teeth filled by modern dentists and had lost all except the one filled with soft gold forty years ago. You could take the gold and pull it out easily.

In regard to amalgam, I think the failure is in the preparation of the cavity, in not getting as perfect a cavity as in the preparation of same for gold. Secondly, I do not believe there is one dentist in twenty five who packs his amalgam into the cavity. I never did it until this spring, and I have been filling teeth for twenty-five years. I experimented on the artificial caries of teeth with fillings and I found that the recurrence of decay of fillings made by different men was nearly the same—very little caries. I have put the teeth into incubator and conducted experiments the same as Dr. Miller has done in his experiments for artificial caries of teeth.

Amalgam, if properly placed in, is a very valuable filling, because there is an antiseptic condition there that prevents the growth of micro organisms in the mouth or production of artificial caries. The therapeutic part of amalgam filling has a very important influence to my mind. Some fifteen or twenty of us packed and filled cavities of teeth for Dr. Black's experiments on the teeth, as we ordinarily did in practice. Each man putting on an amount of pressure as he did in practice, his idea was to find out experimentally the amount of pressure we used in packing a filling. About one and one-half pounds was the average pressure put on the fillings. Dr. Black claims it requires about eight pounds of pressure to pack a perfect amalgam filling; and when this is done there is a shrinkage to the tooth and the cavities were much better filled.

This is an important thing to my mind and one of the possibilities in making good teeth. And I think that Dr. Black very soon demonstrated that most of those who put in amalgam fillings did not use sufficient pressure.

Dr. House, Grand Rapids, Mich.: I have examined the work of a number of dentists in Michigan, and one of the greatest inspirations to me as a young man was in looking over the fillings made by old men. There are fillings in mouths of patients of mine, and in that State, that have been there twenty to twenty-five years. It was my fortune to look over some fillings put in the mouth of a woman twenty-five years ago by a dentist of Detroit.

Dr. Loefler: I wish to make one point. The paper, as I understand it, is this: Concerning the possibilities of filling a tooth. Go on this supposition. Suppose we make a filling now which is perfect, restore the tooth and put it in as good condition as it was originally. If this were possible, would this insure the tooth against any further decay? I want to put that question. Would that insure the tooth against any further decay? If we could reach that high ideal and give it that protection, would that be as much as we could expect? Decay did start there originally.

Dr. Smith: One is possibly of saving tooth and the other the art of saving it.

Dr. Loefler: The point I wish to make is that if we might reach that high degree of skill that Dr. Smith spoke of—if we could reach that skill to make it as approximately as good as that—would that insure the tooth?

Dr. Smith: I purposely avoided discussing failures. I know every man has them, and has his reason for it. To go into discussion of these failures—there is no end of them. My aim was the means by which we might attain the highest degree of the art. Has dentistry advanced in the last century? Yes. Judge it by its best operators. If your filling fails, why is it? Have we exhausted the resources of the art? I want you to look at your perfect fillings. Why do not they save the teeth? Not the poor ones. It is a broad subject, and I confined myself principally to the art. If we reach the highest ideal of the art, whether we use gold or amalgam, can we save the teeth?

Dr. Stephan, of Cleveland: I am glad Dr. Smith has mentioned this last point. I think that to-day we make better fillings than were ever made. These fillings we see to-day are the best of these old operators; we do not see their failures. There are some more rules which have not been touched upon. That is the restoration of contour in bicuspids and molar teeth. I do not think the operator takes enough recognition of this point, so that he will restore the original shape of the tooth and also restore interproximal spaces. I do not think we ought to excuse failure by any lack which we might have in restoring that tooth into its natural shape. How are we sure of that fact? Every man is sure of failure over some work that he has made. When the filling is better the tooth fails.—*Dental Register.*

